# Mast cell tetrahydrobiopterin contributes to itch in mice

**DOI:** 10.1111/jcmm.13999

**Published:** 2018-11-18

**Authors:** Katja Zschiebsch, Caroline Fischer, Annett Wilken‐Schmitz, Gerd Geisslinger, Keith Channon, Katrin Watschinger, Irmgard Tegeder

**Affiliations:** ^1^ Institute of Clinical Pharmacology Goethe‐University Hospital Frankfurt Germany; ^2^ Division of Cardiovascular Medicine University of Oxford Oxford UK; ^3^ Division of Biological Chemistry Biocenter Medical University of Innsbruck Innsbruck Austria

**Keywords:** inflammation, nitric oxide, pain, pruritus, redox signalling, sensory neurons, tetrahydrobiopterin

## Abstract

GTP cyclohydrolase (GCH1) governs de novo synthesis of the enzyme cofactor, tetrahydrobiopterin (BH4), which is essential for biogenic amine production, bioactive lipid metabolism and redox coupling of nitric oxide synthases. Overproduction of BH4 via upregulation of GCH1 in sensory neurons is associated with nociceptive hypersensitivity in rodents, and neuron‐specific GCH1 deletion normalizes nociception. The translational relevance is revealed by protective polymorphisms of GCH1 in humans, which are associated with a reduced chronic pain. Because myeloid cells constitute a major non‐neuronal source of BH4 that may contribute to BH4‐dependent phenotypes, we studied here the contribution of myeloid‐derived BH4 to pain and itch in lysozyme M Cre‐mediated GCH1 knockout (LysM‐GCH1^−/−^) and overexpressing mice (LysM‐GCH1‐HA). Unexpectedly, knockout or overexpression in myeloid cells had no effect on nociceptive behaviour, but LysM‐driven GCH1 knockout reduced, and its overexpression increased the scratching response in Compound 48/80 and hydroxychloroquine‐evoked itch models, which involve histamine and non‐histamine dependent signalling pathways. Mechanistically, GCH1 overexpression increased BH4, nitric oxide and hydrogen peroxide, and these changes were associated with increased release of histamine and serotonin and degranulation of mast cells. LysM‐driven GCH1 knockout had opposite effects, and pharmacologic inhibition of GCH1 provided even stronger itch suppression. Inversely, intradermal BH4 provoked scratching behaviour in vivo and BH4 evoked an influx of calcium in sensory neurons. Together, these loss‐ and gain‐of‐function experiments suggest that itch in mice is contributed by BH4 release plus BH4‐driven mediator release from myeloid immune cells, which leads to activation of itch‐responsive sensory neurons.

## 1 INTRODUCTION

GTP cyclohydrolase 1 (GCH1) is the first‐step, rate‐limiting enzyme in the enzymatic cascade that leads to the generation of the enzyme cofactor, tetrahydrobiopterin (BH4). GCH1 catalyses the hydrolysis of guanosine triphosphate to the intermediate 7,8‐dihydroneopterin‐3′‐triphosphate, which is then further converted to two steps to tetrahydrobiopterin. Tetrahydrobiopterin is essential for the production of biogenic amines via specific hydroxylases, metabolism of phenylalanine,^1^ melanin production in the human skin,^2^ lipid metabolism via alkylglycerol monooxygenase, AGMO^3^ and oxidation/reduction coupling of nitric oxide synthases (NOSs) (Figure S1).^4^, ^5^ Genetic deficiency of GCH1 results in DOPA‐responsive dystonia. The disease manifests with Parkinson‐like motor function deficits owing to the impairment of BH4‐dependent tyrosine hydroxylase activity and hence, shortage in dopamine^6^. Most cells not only utilize de novo produced BH4, but also recycle BH4 from quinoid‐5,6‐BH2 via quinoid dihydropteridine reductase (QDPR). This salvage pathway is used as a source of BH4 for hydroxylases and AGMO^3^ but not NOSs, which recycle BH4 directly within the catalytic domain of the NOS dimer in order to produce NO.^4^, ^7^

Stoichiometric imbalances of NOSs and BH4, which arise from unequal upregulations of NOSs and GCH1,^8^, ^9^ result in redox stress due to the overproduction of hydrogen peroxide (H_2_O_2_) instead of NO.^4^, ^8^ Such imbalances occur at sites of inflammation and at sites of neuronal injury and contribute to hyperexcitability of nociceptive and itching circuits.^10^, ^11^, ^12^ Particularly, inducible NOS (iNOS/NOS2), which is upregulated by subtypes of activated pro‐inflammatory immunocytes, requires high levels of BH4. Owing to the recycling properties, baseline production of serotonin in the brain is fairly maintained in the absence of BH4 de novo synthesis via GCH1.^9^ However, GCH1 ranges among the top 10 of upregulated signalling proteins in human mast cells upon stimulation with IgE^13^ suggesting that BH4 de novo synthesis may become essential for stimulation evoked mediator production in the context of itch.^14^, ^15^ Indeed, serotonin contributes to the activation of itch‐sensitive neurons,^16^ and antigen‐evoked upregulation and phosphorylation of GCH1 with subsequent increase in BH4 has been implicated in protein kinase C dependent degranulation.^17^

Above its functions as a coenzyme, BH4 is released itself^9^, ^18^, ^19^ and may therefore also directly act on nociceptive and itch‐sensitive neurons, for example via redox modification of TRP channels^12^, ^20^, ^21^ or direct stimulation of calcium currents in sensory neurons.^9^ In particular, TRPA1 channels contribute to hydroxychloroquine‐evoked itch,^20^, ^21^, ^22^ and they are redox sensitive.^12^ Tetrahydrobiopterin can act as electron donor and acceptor^1^ and both, relative deficiency and overload, contribute to the pathophysiology of human diseases such as arteriosclerosis and neuropathic pain.^9^, ^23^ Hence, intra‐ and extracellular Tetrahydrobiopterin have the potential to modify channel and receptor properties and impact on neuronal activity in the context of pain and itch. Particularly, activated immune cells, including human peripheral blood monocytes, release BH4 into the extracellular space upon stimulation,^9^ and BH4 injection causes nociceptive hypersensitivity.^9^, ^24^ Therefore, immune cell derived‐BH4 may contribute to pain and itch via two routes (a) indirectly, by regulating mediator production and release and (b) directly by being released itself to act on neighbouring sensory neurons.

To address this hypothesis, we generated mice with myeloid cell specific knockout or overexpression of GCH1. We employed acute and inflammatory nociceptive models as well as Compound 48/80 (Cp48/80) and hydroxychloroquine‐evoked itch models to assess the functions of GCH1/BH4 in and from myeloid immune cells in the context of nociception and itch.

## 2 MATERIALS AND METHODS

### 2.1 Myeloid‐specific GTP cyclohydrolase deficient and overexpressing mice

We generated mice deficient in GCH1, and overexpressing human GCH1 tagged with haemagglutinin (HA), in myeloid cells that express lysozyme M (LysM‐GCH1^−/−^, LysM‐GCH1‐HA) via Cre‐loxP‐mediated recombination. Mice carrying the respective GCH1 LoxP alleles (GCH1‐flfl, GCH1‐HA‐flfl) were mated with mice expressing Cre recombinase under control of the LysM promoter, which is primarily expressed in monocytes, macrophages, mast cells and granulocytes and microglia. The respective floxed mice for knockout and overexpression have been characterized in previous studies addressing the functions of GCH1 in neurons,^25^ Tie2‐expressing macrophages^26^ and vascular cells.^27^ Genotyping was done for the GCH1 floxed allele (primer 5′‐3′: gtccttggtctcagtaaacttgccagg, gcccagccaaggatagatgcag) or GCH1‐HA‐floxed allele (primer 5′‐3′: agttcaggagcgccttacaa, gaacacacccaacattgtgc), and Cre‐recombinase (primer 5′‐3′: gaaagcagccatgtccaatttactgaccgtac, gcgcgcctgaagatatagaaga).

Male and female 8‐15 week old LysM‐GCH1^−/−^ and LysM‐GCH1‐HA and their GCH1‐flfl and GCH1‐HA‐flfl littermates were used for experiments, genotypes matched per groups, with 3 (immunocytochemistry) to 20 (itching) mice per group. The numbers per experiment are outlined in the figure legends and are based on our previous behavioural studies.^28^, ^29^, ^30^ In experiments involving GCH1 inhibition with diamino hydroxypyrimidine (DAHP), we used C57Bl6/J mice, which were randomly allocated to DAHP vs vehicle groups. Behavioural observations and image analyses were done without awareness of genotypes or treatments. Mice had free access to food and water and were housed in climate and light controlled quiet rooms with a 12‐hour light‐dark cycle. General well‐being was assessed by daily inspections and monitoring of body weights (1‐2/week).

The experiments were approved by the local Ethics Committee for animal research (Darmstadt, Germany), adhered to the guidelines for pain research in conscious animals of the International Association for the Study of PAIN (IASP) and those of the Society of Laboratory Animals (GV‐SOLAS) and were in line with the ARRIVE guideline and the European and German regulations for animal research.

### 2.2 Behavioural assessment of itching

Behavioural tests were performed by an investigator, who was not aware of the mouse genotype. Experiments included 8‐20 mice per group. Itching behaviour was measured using two prurigenic substances: Compound 48/80 (Cp48/80, 50 μg in 100 μL saline; Sigma), and hydroxychloroquine (HCQ, 200 μg in 100 μL saline; Sigma) that evoke “histamine‐dependent” and “histamine‐independent” itching responses respectively. Compound 48/80 works by replacing histamine and by stimulation of degranulation,^31^ hence the Cp48/80 “release‐soup” also contains other mediators of pre‐formed mast cell granula.^32^ Compound 48/80 or HCQ was injected intradermally at the nape of the neck and the scratching response was observed for 30 minutes starting immediately after injection. The time the mouse spent scratching the site of injection was recorded with a stopwatch and the “scratching time” is used as quantitative metric readout. In experiments involving GCH1 inhibition, DAHP (150 mg/kg in 50% PEG/PBS) or vehicle was injected intraperitoneally 30 minutes prior to Cp48/80 or HCQ, and scratching time was measured as described above. The dose of DAHP is based on previous studies.^9^, ^33^ It has no adverse effects, does not alter motor functions, activity or “depression‐like” behaviour.^9^, ^24^, ^33^

### 2.3 Behavioural assessment of inflammatory nociception

For the chronic inflammatory model 20 μL of complete Freund's adjuvant (CFA) was injected into the plantar side of the left hind paw. The formalin test was initiated by injecting 20 μL of 5% formalin into the dorsal side of one hind paw. The time the mouse spent licking the injected paw was recorded for 45 minutes with a stopwatch starting immediately after formalin injection. Test results were expressed as time course and the total licking times.

For assessment of thermal and mechanical nociception, mice were habituated for three consecutive days to the test room, test cages and set‐ups. The latency of paw withdrawal on mechanical stimulation was assessed with a dynamic von Frey apparatus (Aesthesiometer, Ugo Basile, Italy) employing a force range of 0‐5 g, ramp of 0.5 g/s and hold at 5 g until paw withdrawal. The sensitivity to painful heat stimuli was assessed by recording the paw withdrawal latency in the Hargreaves test (IITC Life Science), which employs a radiant heat source placed underneath the hind paw with a mirror system. The lamp emits a heat beam until the paw is withdrawn. Behavioural tests were performed at baseline and up to 2‐3 weeks after paw inflammation.

### 2.4 In vivo imaging of paw inflammation

In vivo imaging of paw inflammation was done with an IVIS Lumina Spectrum as described^34^ and bioluminescence signals were analysed with Living Image software (Perkin Elmer). Fourteen days after induction of paw inflammation, 150 μL XenoLight RediJect Inflammation Probe (40 mg/mL; PerkinElmer) that recognizes myeloperoxidase activity was injected intraperitoneally and bioluminescence of the hind paw was captured 5, 10 and 15 minutes after injection in eight animals per genotype. During the imaging procedure, mice were kept under 1%‐1.5% isoflurane anaesthesia. The IVIS settings were as follows: Epi‐bioluminescence, emission filter open, excitation filter block, fstop 1, binning 8, focus B 6.5 cm, exposure 60 seconds. Regions of interest (ROI) that is the ipsilateral paws were set to software‐aided automatic detection. The contralateral paw served as control. For each mouse, the two maximum time points of the total counts of bioluminescence in ROIs were used for statistical analysis of genotype differences.

### 2.5 Peritoneal macrophages

To harvest peritoneal macrophages (PMs) mice were injected with 1 mg/kg lipopolysaccharide (LPS) intraperitoneally. At 24 hours after injection, the peritoneal cavity was flushed with ice‐cold PBS and cells were collected and pelleted at 405 *g* for 3 minutes at 4°C. Cells were incubated with erythrocyte lysis buffer (150 mmol/L NH_4_CL, 100 mmol/L NaHCO_3_, 0.1 mmol/L Na‐EDTA, pH 7.4) for 10 minutes and subsequently centrifuged and washed with PBS. After resuspension in RPMI medium supplemented with 200 mmol/L GlutaMax, 10% foetal calf serum (FCS), 1% penicillin/streptomycin, 0.1 mmol/L nonessential amino acids and 50 μmol/L 2‐mercaptoethanol, PMs were seeded in 12‐well plates on cover slips. Cells were kept at 37°C at 5% CO_2_ for 4 hours to become adherent. Non‐adherent cells were discarded and adherent macrophages were used for immunofluorescence studies.

### 2.6 Primary bone marrow derived mast cells and macrophages

Femur and tibia were flushed with 1× PBS supplemented with 0.5% penicillin/streptomycin. Cells were collected by centrifugation (405 *g*, 3 minutes, 4°C), and incubated for 10 minutes in erythrocyte lysis buffer (150 mmol/L NH_4_CL, 100 mmol/L NaHCO_3_, 0.1 mmol/L Na‐EDTA, pH 7.4). After washing, cells were resuspended and seeded in bone marrow derived mast cell (BMMC) or macrophages (BMDM) medium. BMMCs medium consisted in RPMI 1640 supplemented with 10% foetal bovine serum, 25 mmol/L HEPES, 4 mmol/L L‐glutamine, 0.1 mmol/L nonessential amino acids, 1 mmol/L sodium pyruvate, 50 μmol/L 2‐mercaptoethanol, 100 U/mL penicillin, 100 μg/mL streptomycin, 10 ng/mL recombinant murine interleukin‐3 (IL‐3; PeproTech) and recombinant murine stem cell factor (SCF; PeproTech). Cells were differentiated for 4‐5 weeks and kept at 37°C at 5% CO_2_. Medium was exchanged every 3‐4 days with freshly added IL‐3 and mSCF. The differentiation towards BMMCs was controlled by FACS analyses (BD FACS Canto II) with lineage‐specific fluorochrome labelled antibodies (CD117‐PE, FcεRIα‐FITC, Table S1).

For preparation of macrophages, cells were seeded in RPMI 1640 supplemented with 200 mmol/L GlutaMax, 10% FCS, 100 U/mL penicillin, 100 μg/mL streptomycin and 20 ng/mL macrophage colony stimulation factor (M‐CSF) to initiate differentiation towards mature macrophages within 7 days. Cells were kept at 37°C at 5% CO_2_. Differentiated BMDMs were stimulated for 24 hours with 2 mmol/L DAHP, 0.1 mmol/L BH4 and 1 μg/mL LPS + 50 ng/mL IFNγ or vehicle (0.1% DMSO) in cell culture medium without M‐CSF and were used for analysis of mRNA and protein expression, oxidative parameters (H_2_O_2_, NO) and concentration of pterins. The concentrations of the treatments were based on our previous studies.^9^, ^33^, ^35^

### 2.7 Analysis of mast cell degranulation, histamine and serotonin release

Differentiated BMMCs were used for measurements of histamine and serotonin levels and mast cell degranulation using ELISA based assays. After 4 (histamine)—5 weeks (serotonin) of differentiation, BMMCs were collected as single cell suspensions, pelleted and lysed in Tyrode's buffer (130 mmol/L NaCl, 5 mmol/L KCl, 10 mmol/L HEPES, 5.6 mmol/L glucose, 1.4 mmol/L CaCl_2_, 1 mmol/L MgCl_2_, 0.1% BSA, pH 7.4) containing 2% Triton‐X100 for 30 minutes. Subsequently, cells were lysed with ultrasound, centrifuged at 16 168 *g* for 20 minutes and supernatants containing the pruritic amines were used for the respective ELISA. The histamine (#BA E‐1000; Labor Diagnostika Nord) and serotonin (#BA‐E 5900; Labor Diagnostika Nord) assays were carried out according to the manufacturers’ instructions. Briefly, samples were acetylated for 30‐45 minutes, subsequently incubated with the respective antiserum overnight at 4°C, and then treated with the enzyme conjugate for 30 minutes. Subsequently, substrates were added to the wells and incubated for another 20‐30 minutes in the dark. Finally, after stopping the reaction, absorbance was immediately measured using a microplate reader set to 450 nm with a reference wavelength of 620 nm.

For mast cell degranulation a tryptase‐based assay was used (#IMM001; Merck Millipore), which is based on spectrophotometric detection of the chromophore p‐nitroaniline (pNA) after cleavage from the labelled substrate tosyl‐Gly‐Pro‐Lys‐pNA. The free pNA can then be quantified using a spectrophotometer. BMMCs were freshly prepared after 4 weeks of differentiation. To induce degranulation in BMMCs, each 1 × 10^6^ cells were stimulated with 500 nmol/L calcium ionophore and incubated for 1 hour at 37°C. Cells were centrifuged at 405 *g* for 5 minutes and supernatants were collected and stored at 4°C. Cell pellets were resuspended in assay buffer, sonified, centrifuged at 405 *g* for 20 minutes and the supernatant used for analysis. Preparation of samples, pNA control and tryptase control were conducted according to the assay instructions. After adding tryptase substrate to each well to initiate the colorimetric reaction, the 96‐well plate was incubated for 1‐2 hours and absorbance was measured at 450 nm using a microplate reader.

### 2.8 Quantitative real‐time PCR

Total RNA was extracted from BMDMs according to standard procedures using TRI reagent, and was reverse transcribed using poly‐dT as a primer to obtain cDNA fragments. Quantitative real‐time PCR (QRT‐PCR) was performed on an ABI 7500 Fast Real‐time PCR System (Applied Biosystems, Darmstadt, Germany) using the SYBR green technique (Maxima SYBR Green/ROX qPCR Master Mix; Thermo Fisher Scientific). Transcript regulation relative to the housekeeping gene, PPIA was determined using the relative standard curve method according to the manufacturer's instructions (Applied Biosystems). Amplification was achieved at 60°C for 35 cycles.

### 2.9 Western blot analysis

Whole cell protein extracts were prepared in RIPA lysis buffer (Cell Signalling) containing a protease inhibitor cocktail (Complete™; Roche Diagnostics, Mannheim, Germany) and PMSF 10 μg/mL. Tissue samples were homogenized in PhosphoSafe Buffer (Sigma) enriched with 10 μmol/L Pefabloc (serine‐protease inhibitor). Proteins were separated by 12% SDS‐PAGE, transferred to nitrocellulose membranes (Amersham Pharmacia) by wet‐blotting and detected using the anti‐human GCH1 (Sigma) and secondary antibodies conjugated with IRDye 680 or 800 (1:10 000; LI‐COR Biosciences, Bad Homburg, Germany). Beta‐actin was used as a loading control. Antibodies are listed in Table S1. Blocking was achieved with 5% skimmed milk in 0.1 Tween 20 in 1× PBS. All incubations were done in Tris‐buffered saline containing 0.1% Tween 20 or in Odyssey buffer. Blots were visualized and analysed on the Odyssey Infrared Imaging System (LI‐COR Biosciences), quantified with Image Studio Lite (LICOR Biosciences) and the ratio of the respective protein band to the control band was used as semi‐quantitative readout.

### 2.10 Immunofluorescence of macrophages and mast cells

Cell culture slides were washed and fixed with 4% paraformaldehyde (PFA) for 20 minutes. After washing with 1× PBS and permeabilization with 0.1% Triton‐X100 in PBS (PBST), cells were blocked in 3% BSA in PBST for 1 hour and subsequently incubated overnight with anti‐human GCH1 (1:200; Abnova) or anti‐HA‐tag (for OE cells) in 1% BSA in PBST at 4°C. After washing, cells were incubated with the secondary antibody coupled to Alexa Fluor 488 (1:800; Thermo Fisher Scientific) for 2 hours at room temperature. Subsequently, slides were incubated with CD11b (Serotec) overnight at 4°C and treated with a secondary Cy3 labelled antibody (1:800; Sigma). To visualize cell nuclei, DAPI staining was performed for 10 minutes in 1× PBS and slides were mounted in Aqua Poly/Mount medium (Polysciences GmbH, Eppelheim, Germany). Antibodies are listed in Table S1.

Bone marrow derived mast cell‐suspension cells were stained in 1.5 mL Eppendorf tubes using CD117‐PE and FcεRIα‐FITC primary and secondary antibodies (Table S1). Stained BMMCs were then pelleted onto cover slips in a 12‐well plate, and cover slips were mounted with Aqua Poly/Mount medium. For microscopy, we used an inverted fluorescence microscope (Zeiss Axiovert 200) equipped with AxioVision 4.9 software (Zeiss).

### 2.11 Histology and tissue immunofluorescence

Terminally anaesthetized mice were perfused transcardially with 0.9% saline, followed by 4% PFA in 1× PBS for fixation. Liver, kidney, lung and skin were excised, post‐fixed in 4% in 1× PBS for 2 hours, transferred into 20% sucrose in 1× PBS for overnight cryoprotection at 4°C and subsequently imbedded in Tissue‐Tek^®^ O.C.T. Compound (Science Services, Munich, Germany). Transverse sections were cut on a cryotome (12 μm), mounted on glass slides, which were subjected to immunofluorescence analyses using antibodies and procedures described above. Skin sections were cut on a cryotome (12 μm). Cryosections were permeabilized with 0.1% Triton‐X100 in PBS (PBST), blocked in 3% BSA or blocking reagent in PBST for 1 hour and incubated overnight with the (first) primary antibody in 1% BSA or blocking reagent in PBST at 4°C. After washing, sections were incubated with the secondary antibody coupled to Alexa Fluor 488 or Cy3 for 2 hours at room temperature. In case of two‐colour stainings, the procedure was repeated with the next primary‐secondary pair. Slides were finally washed and mounted in Aqua Poly/Mount medium (Polysciences GmbH, Eppelheim, Germany). Microscopic tissue analyses were performed with a Keyence (BZ‐9000) inverted microscope with automatic stitching.

ImageJ was used for quantification of immunoreactive cells or particles. RGB images were separated into the respective channels. Single channel 8bit images were background subtracted, and the threshold was set using the Isodata algorithm implemented in ImageJ. Subsequently immunoreactive particles were counted with the Particle Counter in ROI, which were set to exclude regions of immunoreactivity of hair roots. The analysis was done with sections of three mice per group. Results are presented as number of particles. For analysis of GCH1 immunoreactivity in macrophages, ROIs were defined representing individual cells. After setting thresholds according to the Yen algorithm (ImageJ), the particle counter was used to measure the percent area covered by GCH1 immunofluorescence.

Immunostainings of human paraffin embedded tissue arrays (Human Skin AccuMax T212) followed the manufacturer's instructions. Briefly, sections were deparaffinized in xylene and graded ethanol and antigen retrieval was achieved with 10 mmol/L citrate buffer, pH 6.0 containing 0.05% Tween 20. Incubation with the primary anti‐human GCH1 antibody (Abnova) was done in PBST with 10% serum overnight at 4°C. Subsequently, slides were incubated with an Alexa‐488 labelled secondary antibody (2 hours RT), finally washed and cover slipped with Fluoromount. Exemplary images of H&E stains of the skin sections were taken as provided by the manufacturer.

### 2.12 Analysis of pterins

Biopterin and neopterin concentrations in plasma or in BMDMs cells were analysed with liquid chromatography coupled with tandem mass spectrometry (LC‐MS/MS) as described.^33^ Unstimulated cells or cells stimulated for 16 hours with 50 μmol/L forskolin (Sigma), were incubated with vehicle (0.1% DMSO), 2 mmol/L DAHP (IC50 for GCH1 inhibition is 2‐3 mmol/L) and 0.1 mmol/L BH4 for 3 hours. Cells were scraped off, counted and pelleted. The cell pellet was used for extraction and analysis of biopterin and neopterin. The supernatants were immediately used for H_2_O_2_ and NO measurements with standard enzyme assays.

Briefly, pteridines were obtained after acidic oxidation of homogenized cells or plasma samples by solid phase extraction using Oasis MCX extraction cartridges (Waters GmbH, Eschborn, Germany) after spiking samples with the internal standard rhamnopterin. HPLC analysis was done under gradient conditions using a Gemini C18 5 μm, 150 × 2 mm column (Phenomenex, Aschaffenburg, Germany). MS/MS analyses were performed on an API 4000 Q TRAP, a hybrid triple quadrupole/linear ion trap mass spectrometer with a turbo ion spray source. Precursor‐to‐product ion transitions of m/z 236→192 for biopterin, m/z 252→192 for neopterin and m/z 266→192 for rhamnopterin were used for the MRM. Quantification was done with Analyst software 1.5 (AB Sciex). Linearity of the calibration curve was proven from 0.1 to 100 ng/mL. The intra‐day and inter‐day variability were <10%.

### 2.13 Amplex Red assay for hydrogen peroxide

The Amplex Red assay (Life Technologies) was used to determine levels of H_2_O_2_ according to the manufacturer's instructions. Twenty‐four hours after stimulation, the supernatants of 50 000 cells per well were incubated with 50 μL Amplex^®^ Red substrate for 30 minutes in the dark at room temperature, followed by fluorescence measurements at 540/595 nm excitation/emission on the Spectra Fluor Plus^®^ instrument with XFluor^®^ software (Tecan, Crailsheim).

### 2.14 Saville‐Griess assay of nitric oxide

The concentration of nitrite/nitrate was determined with the Saville‐Griess assay adapted for microtiter plates. A standard curve was prepared with serial dilutions (0‐50 μmol/L) of a freshly prepared sodium nitrite (NaNO_2_) stock solution (100 mmol/L). Two hundred microlitre of the collected BMDM supernatants was added to a well of a 96‐well plate. Fifty microlitre of sulphanilamide solution (4 mg/mL in 1 N HCl) was added to standards and samples. After short incubation of 1‐2 minutes, 50 μL of *N*‐(naphthyl)‐ethylenediamine dihydrochloride solution (6 mg/mL in H_2_O) was added followed by incubation for 5 minutes at room temperature and measuring absorbance at 540 nm with a Spectra Fluor Plus^®^ instrument and XFluor^®^ software (Tecan).

### 2.15 Primary neuron culture

Primary adult dissociated DRG neuron‐enriched cultures were prepared by dissecting mouse DRGs into HBSS (Ham's balanced salt solution, Dulbecco) and 10 mmol/L HEPES, followed by digestion with 5 mg/mL collagenase A and 1 mg/mL dispase II (Roche Diagnostics) prior to treatment with 0.25% trypsin (GibcoBRL, Karlsruhe, Germany). Triturated cells were centrifuged through a 10% BSA solution prior to plating on poly‐l‐lysine and laminin coated cover slips in Neurobasal medium (GibcoBRL) containing 2% (vol/vol) B27 supplement (GibcoBRL), 50 μg/mL Pen‐Strep, 100 ng/mL NGF and 200 mmol/L L‐glutamine. After incubation for 2 hours, 2 mL Neurobasal medium was added and neurons were incubated for 2 days or 7 days depending on the experiment with half exchange of the medium at 3 days. Cells were kept at 37°C, 5% CO_2_, 95% humidity.

### 2.16 Calcium Imaging

Cultured adult DRG neurons were loaded for 1 hour with 10 μmol/L fura‐2 (Invitrogen) in Neurobasal medium with 0.02% pluronic (w/v). The temperature was kept at 37°C throughout the measurements. The neurons were continuously perfused with Ringer solution (in mmol/L: 136 NaCl, 5.4 KCl, 1.8 CaCl_2_, 1 MgCl_2_, 0.33 NaH_2_PO_4_, 10 Glucose, 10 HEPES) at a speed of 2 mL/min. Images were captured at a rate of 1 frame per 2 seconds with a high speed camera. Intracellular [Ca^2+^]i was assessed fluorimetrically as absorbance ratio at 340 and 380 nm excitation (F340/380) (510 nm for emission). Baseline ratios were recorded for 100 seconds before bath application of H_2_O_2_ (1 mmol/L), DEA‐NO (500 μmol/L) or BH4 (100 μmol/L). The stimulation time was 100 seconds. After a washout period, cells were perfused with high potassium (50 mmol/L KCl in Ringer) to check the viability of the neurons. Only viable neurons were included in the analysis, the KCl‐evoked peak is not shown. Data are presented as changes in the fluorescence ratios (F340/F380) normalized to baseline ratios. The peak fold change of [Ca^2+^]i and the area under the “fold change vs time curves” (AUCs) were used for statistical comparisons.

### 2.17 Statistical analyses

Data are presented as mean ± SD unless stated otherwise in the respective figure legend and were analysed with SPSS 23 or 24 and Graphpad Prism 6.0. Data were submitted to univariate analysis of variance (ANOVA) or unpaired, two‐tailed Student's *t* tests, the latter in case of two groups. Time course data were submitted to analysis of variance for repeated measurements (rm‐ANOVA) using the within subject factor “time” and the between subject factor “genotype” or “group.” Sphericity was assessed with Mauchly's test, and if violated (Mauchly's *P* < 0.05), degrees of freedom were adjusted according to Huynh Feldt. Areas under the behaviour vs time curves (AUC) were calculated with the linear trapezoidal rule and submitted to univariate ANOVA. In case of significant differences of ANOVAs, groups were compared with the respective control groups using t tests with a correction of alpha according to Dunnett (one control group) or according to Šidák‐Bonferroni (specific control groups). The alpha level was set to 0.05 for all comparisons, and adjusted *P*‐values are reported.

## 3 RESULTS

### 3.1 Myeloid‐specific deletion and overexpression of GCH1

**Figure 1:**
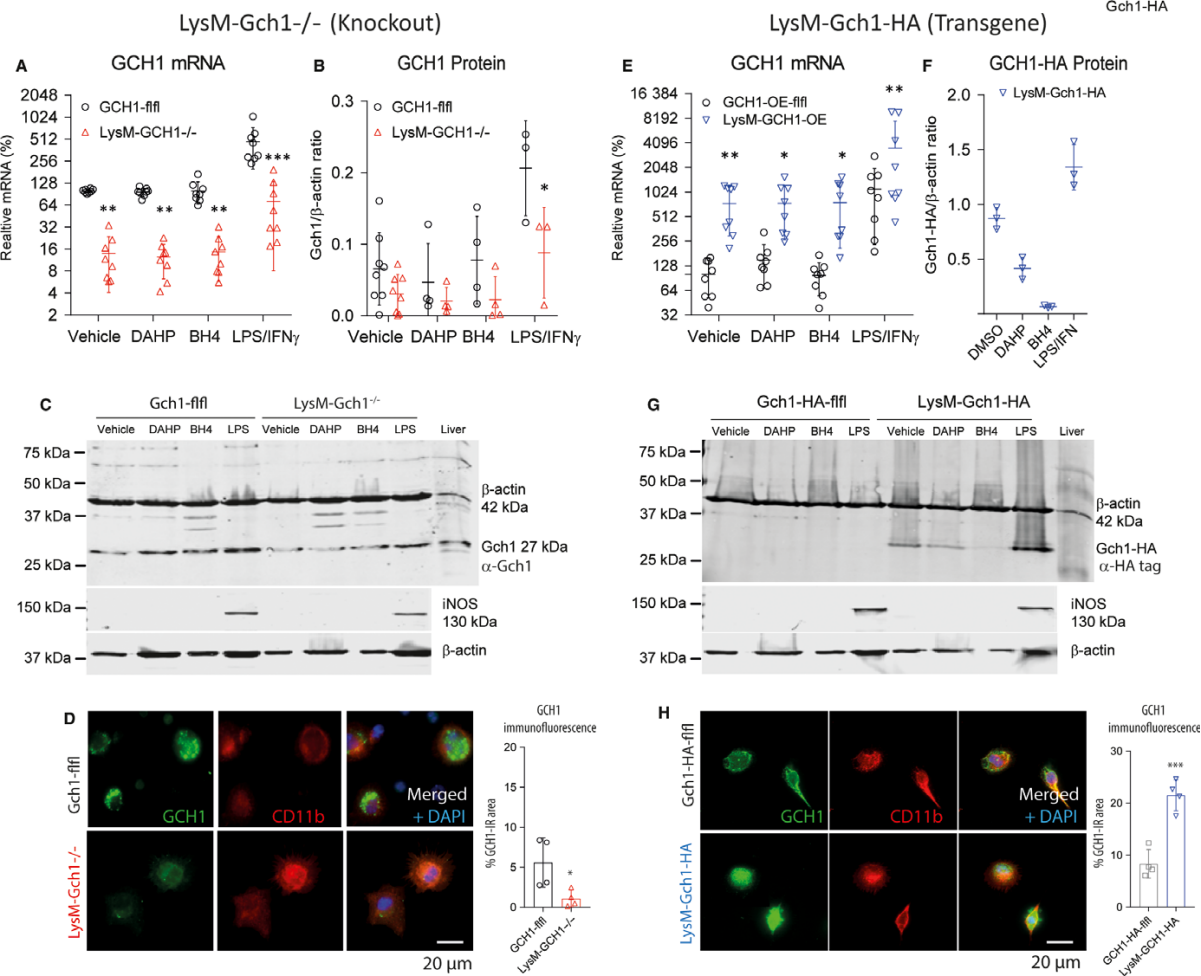
Expression and regulation of GTP cyclohydrolase 1 (GCH1) in lysozyme M (LysM) driven, myeloid cell specific knockout (left panel) and transgenic overexpressing mice (right panel). A‐C, Quantitative RT‐PCR and Western blot analysis of GCH1 expression in bone marrow derived macrophages (BMDMs) of LysM‐GCH1^−/−^ (knockout) and floxed control mice. Mice were treated for 24 h with vehicle (= DMSO 1%), 2 mmol/L of the GCH1‐inhibitor, DAHP (diaminohydroxypyrimidine), 100 μmol/L BH4 or 1 μg/mL LPS + 50 ng/mL IFNγ. RT‐PCR data are the mean and SD of n = 8 mice per group and two cultures per condition. The Western blot in (C) shows an exemplary result. Liver was used as control tissue. Quantitative protein data are from n = 3‐8 mice per group, shown in (B). Uncut blots are shown in Figure S2A. Further blots are shown in Figure S2B. D, Immunofluorescence analysis of GCH1 in peritoneal macrophages of LysM‐GCH1^−/−^ and floxed control mice. CD11b was used as marker for stimulated macrophages. The right scatter‐bar plot shows the quantification of GCH1 immunofluorescence expressed as percent GCH1‐immunoreactive area in ROIs, defined by the cell circumferences (* *t* test, *P *<* *0.05). E–H, In analogy to the left panel the right‐panel graphs show the quantitative RT‐PCR, Western blot and immunocytology of GCH1 expression of transgenic LysM‐GCH1‐HA and floxed control mice. The LysM‐GCH1‐HA blot was developed with anti‐HA‐tag antibody. Therefore, there is no band in the floxed control mice. Asterisks indicate significant differences between genotypes (two‐way ANOVA, subsequent *t* test per stimulation, Holm‐Šidák adjustment, or *t* test for comparisons of two groups)

**Figure 2:**
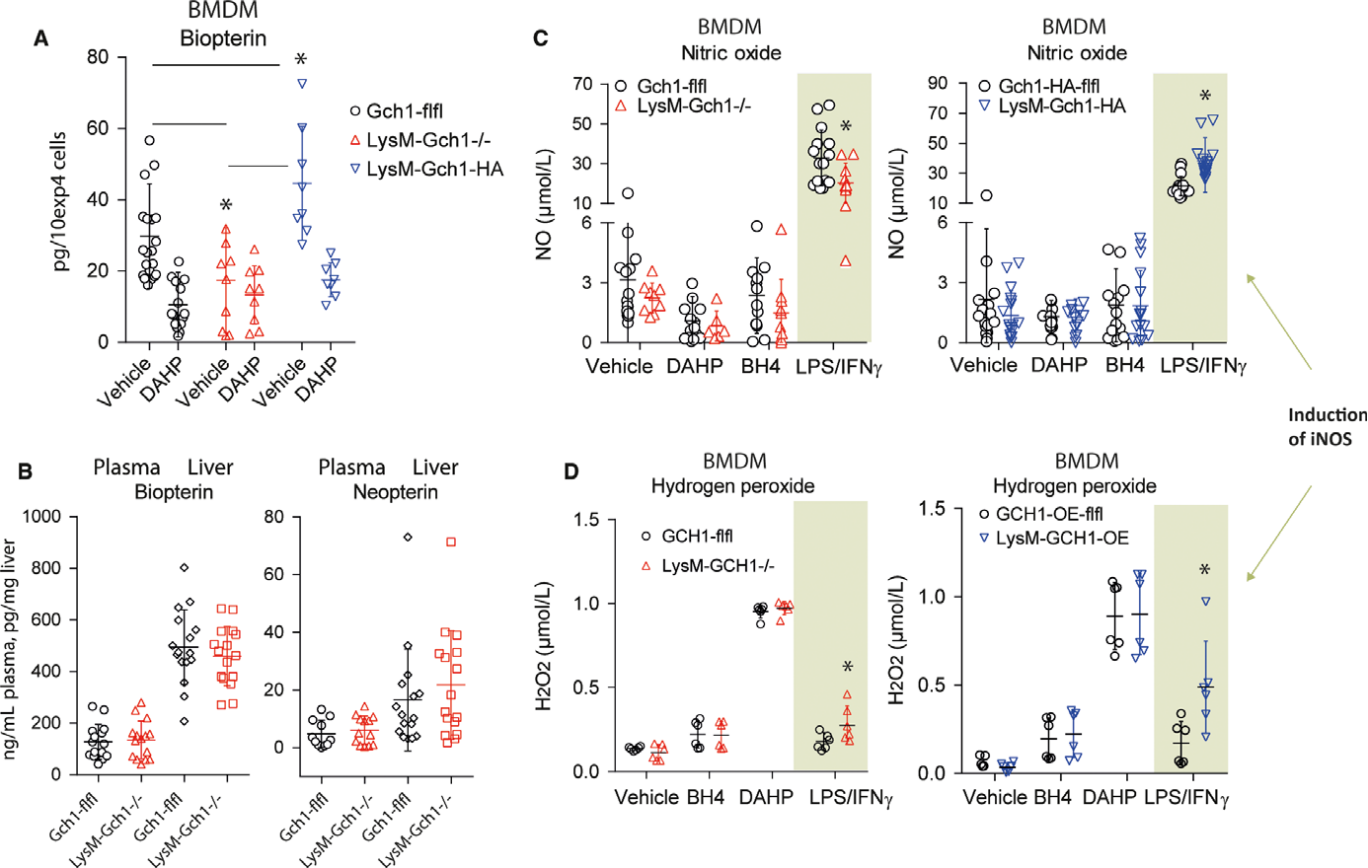
A, Scatter plots of biopterin concentrations in BMDM‐lysates in LysM‐GCH1^−/−^, LysM‐GCH1‐HA and the respective floxed control mice treated with vehicle of DAHP (2 mmol/L for 24 h). Each scatter represents a mouse, the line is the mean and whisker show the SD. Asterisks indicate significant differences between genotypes as indicated (two‐way ANOVA, subsequent *t* test per stimulation, Holm‐Šidák adjustment; *P <* *0.05). B, Control experiment showing unaltered systemic biopterin and neopterin concentrations in plasma and liver tissue homogenates in LysM‐GCH1^−/−^ vs floxed control mice. Each scatter represents a mouse. C and D, Nitric oxide and hydrogen peroxide (H_2_O_2_) concentrations in BMDM‐supernatants of LysM‐GCH1^−/−^, LysM‐GCH1‐HA and the respective floxed control mice treated for 24 h with vehicle, BH4 (100 μmol/L), DAHP (2 mmol/L) or stimulated with 1 μg/mL LPS + 50 ng/mL interferon gamma (IFNγ). The concentrations of the treatments are based on our previous studies.^9^, ^24^ Asterisks indicate significant differences between genotypes (two‐way ANOVA, subsequent *t* test per stimulation, Holm‐Šidák adjustment; *P <* *0.05; H_2_O_2_ data are of n = 6 mice per group)

Lysozyme‐Cre leads to specific excision of loxP‐flanked sequences in cells of myeloid origin.^36^ We confirmed the expected myeloid‐specific knockout (Figure 1A‐D) or overexpression (Figure 1E‐H) of GCH1 in BMDMs at mRNA, protein (Figure 1) and product levels (Figure 2).

RT‐PCR revealed about 90% reduction of GCH1 mRNA in LysM‐GCH^−/−^ BMDMs as compared to GCH1‐flfl BMDMs, which were set to 100% (Figure 1A). Oppositely, LysM‐GCH1‐HA BMDMs had about 10‐fold higher (1000% higher) mRNA levels than the respective floxed controls (Figure 1E). The expression differences were confirmed at the protein level per Western blots (Figure 1B, F quantification; Figure 1C, G examples) using anti‐GCH1 antibody for LysM‐GCH^−/−^ BMDMs (Figure 1B, C) and anti‐HA‐tag in LysM‐GCH1‐HA BMDMs (Figure 1F, G). Tetrahydrobiopterin suppressed the expression of the transgene, likely a feedback regulation. Immunofluorescence analyses (Figure 1D, H) identified BMDMs via CD11b, and showed the expected suppression (LysM‐GCH^−/−^) vs increase (LysM‐GCH1‐HA) of GCH1 in these cells.

In myeloid‐derived immune cells, BH4 is important as coenzyme of inducible nitric oxide synthase (iNOS), which produces NO and H_2_O_2_, the latter depending on the stoichiometric balance of iNOS and BH4. Tetrahydrobiopterin is also needed for AGMO and hydroxylases, but these enzymes are less dependent on de novo synthesis because they can use BH4, which was recycled from BH2 via QDPR. Therefore, we primarily assessed biopterin levels, NO and H_2_O_2_ for functional assessment of the knockout and overexpression. Biopterin analyses show that BH4 is reduced, but still available at low levels in LysM‐GCH1^−/−^ BMDMs (Figure 2A), likely maintained through recycling from BH2. Inversely, LysM‐GCH1‐HA BMDMs have increased total biopterin, which is only partly suppressed by DAHP at its IC50 concentrations (Figure 2A). A control experiment showed that biopterin and neopterin (inactive GCH1‐derived byproduct) levels in plasma and liver were normal in LysM‐GCH1^−/−^ mice (Figure 2B), showing that systemic GCH1 was not affected by lysozyme Cre‐mediated deletion as expected. Also in line with the expectations, NO levels were very low in non‐stimulated cells in all BMDMs, no matter if GCH1 was knocked out or overexpressed (Figure 2C), owing to the low baseline expression of iNOS (Figure 1C and G) and hence low baseline de novo demand of BH4. However, after stimulation with LPS/IFNγ, used as a pro‐inflammatory stimulus, LysM‐GCH1^−/−^ BMDMs produced significantly less NO than control cells (Figure 2C). Oppositely, LysM‐GCH1‐HA BMDMs produced more NO (Figure 2C) and more H_2_O_2_ (Figure 2D) than the controls. The stimulation also resulted in an increase of H_2_O_2_ in the knockouts (Figure 2D), owing to the uncoupling of the catalytic oxidation‐reduction cycling in iNOS if BH4 is missing.

### 3.2 Inflammatory nociception

**Figure 3:**
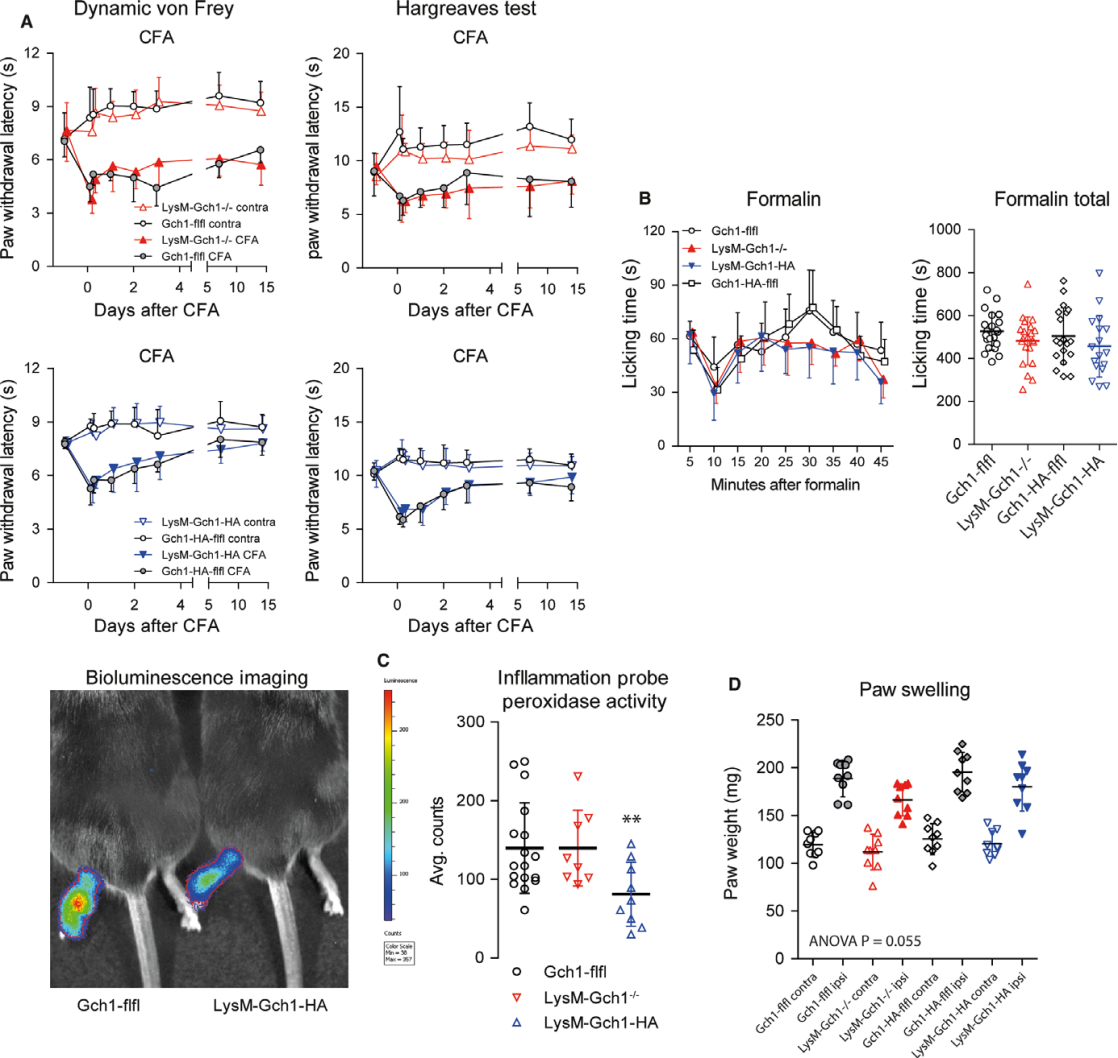
Nociceptive behaviour of myeloid cell specific GTP cyclohydrolase 1 (GCH1) knockout and overexpressing mice in the complete Freund's adjuvant induced paw inflammation model and formalin test. A, Paw withdrawal latencies on mechanical and heat stimulation of the ipsilateral and contralateral hind paws before and after induction of CFA‐evoked paw inflammation in LysM‐GCH1^−/−^ and GCH1‐flfl mice (upper panel, knockout) and in LysM‐GCH1‐HA and GCH1‐HA‐flfl mice (bottom, overexpression). For mechanical stimulation, a dynamic von Frey plantar test was used. Heat sensitivity was assessed with the Hargreaves test. The data are means ± SD of n = 9 mice per group. B, Time course of formalin induced paw licking behaviour (left) and total licking time (right) in LysM‐GCH1^−/−^ and GCH1‐flfl mice, and in LysM‐GCH1‐HA and GCH1‐HA‐flfl mice (n = 17 mice per group, means ± SD). C, In vivo visualization and quantification of peroxidase activity in the CFA inflamed hind paw in LysM‐GCH1^−/−^ and GCH1‐flfl mice, and in LysM‐GCH1‐HA and GCH1‐HA‐flfl mice using a peroxidase‐sensitive bioluminescent substrate (Inflammation Probe). Scatter plots show results of individual mice (n = 9), the line is the mean and whiskers show the SD. LysM‐GCH1‐HA differed significantly from the other groups (**P <* *0.01; one‐way ANOVA, posthoc *t* tests employing a Šidák adjustment for multiple testing). D, Scatter plots showing the weight of the ipsilateral and contralateral hind paws at the end of the behavioural analysis in the CFA evoked paw inflammation model (n = 9 per group). The paw weight reflect the inflammatory paw swelling and oedema. Data are results of individual mice, the line is the mean and whiskers show the SD

We assessed inflammatory nociception in the CFA induced paw inflammation model. As expected, heat and mechanical paw withdrawal latencies dropped after CFA injection and recovered over time. There was no difference between genotypes, neither for LysM‐mediated GCH1 knockout (Figure 3A, red) nor for GCH1 overexpression (Figure 3A, blue). There was also no difference in formalin‐evoked paw licking behaviour (Figure 3B) but LysM‐mediated GCH1 overexpression reduced peroxidase activity in the inflamed paw as assessed by in vivo bioluminescence imaging of a peroxidase‐sensitive probe (Figure 3C), whereas GCH1 knockout had mildly weaker inflammatory paw swelling (Figure 3D). The result points to dual inflammation‐relevant functions of myeloid GCH1/BH4. On the one hand, it drives iNOS activity with production of NO and H_2_O_2_, which is characteristic for pro‐inflammatory macrophages (M1‐like), but on the other hand it also allows for AGMO‐dependent M2‐like polarization,^37^ hence resolution of inflammation.

### 3.3 Pruritogen‐evoked scratching responses

Previous studies showed that not only human monocytes but also mast cells upregulate GCH1/BH4 upon stimulation,^13^ and pointed at BH4‐dependent mechanisms of mast cells degranulation and mediator release.^38^ To assess the in vivo relevance of these functions, we assessed behaviour in two models of itch, the Compound 48/80 evoked histamine release‐model and hydroxychloroquine‐evoked histamine‐independent itch.^39^ Compound 48/80 causes histamine plus other mediator release by vesicular exchange of histamine vs Cp48/80^32^ and by activation of Mas‐related G‐protein coupled receptor, MrgprB2 (MRGB2) of mast cells.^40^ It also directly activates^40^ TRPV4 channels.^41^ Hydroxychloroquine on the other hand, activates Mas‐related G‐protein coupled receptors, MrgprA3^42^, ^43^, ^44^ and TRPA1 channels,^21^ and is considered to represent a model of “histamine‐independent” itch, but chloroquine also causes a release of classical mediators and arachidonic acid derived lipids from mast cells.^45^, ^46^

**Figure 4:**
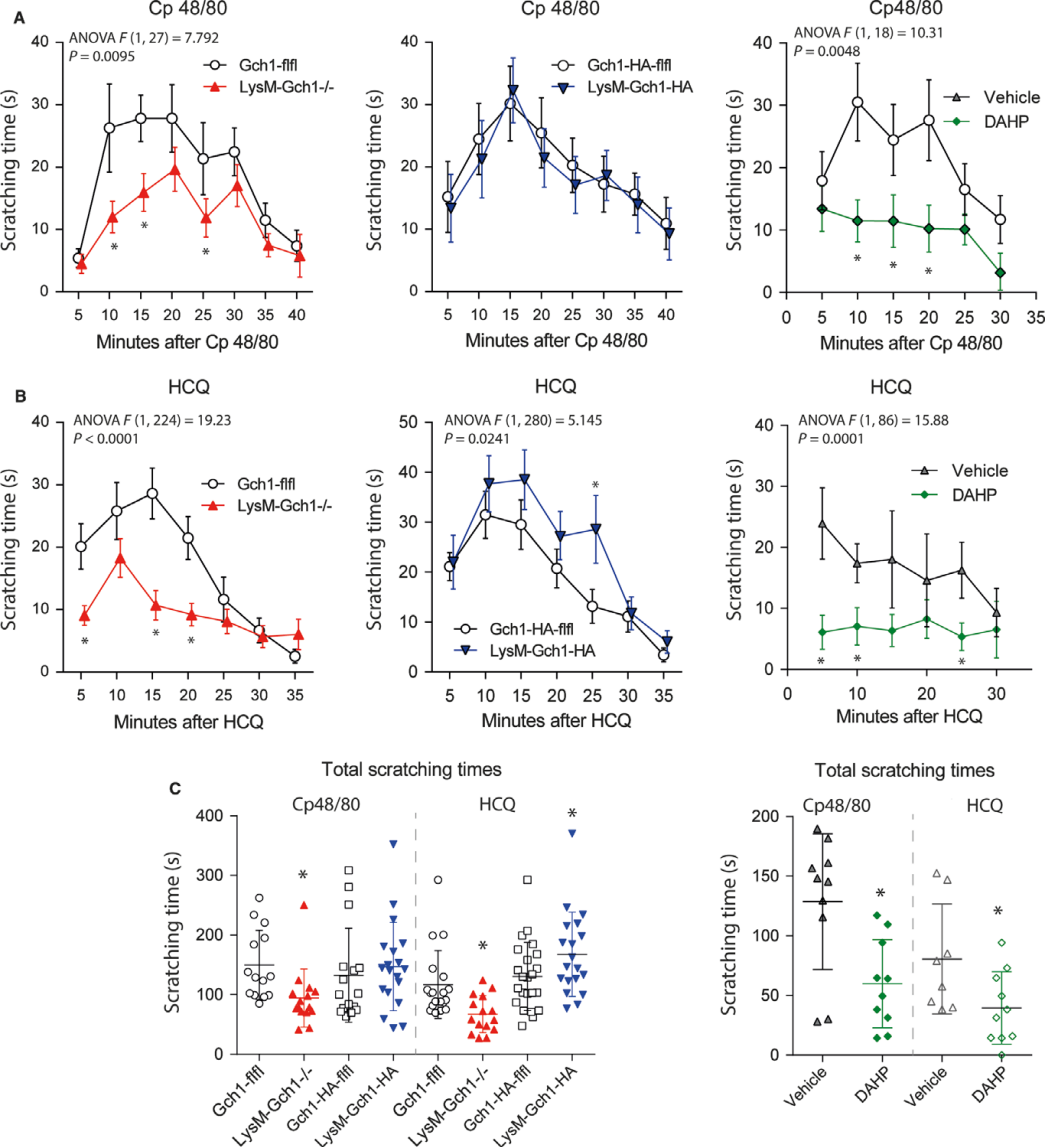
Scratching behaviour of myeloid cell specific GTP cyclohydrolase 1 (GCH1) knockout and overexpressing mice or wild‐type mice treated with DAHP in Compound 48/80 (Cp48/80) and hydroxychloroquine (HCQ) evoked itching models. A, Time courses (mean ± SD) of the scratching times after dermal injection of Cp48/80, which causes histamine release in the skin. Data are of n = 14‐19 mice per genotype and n = 10 for DAHP (150 mg/kg) vs vehicle. LysM‐GCH1^−/−^ showed significantly less scratching behaviour than GCH1‐flfl mice. Similarly, DAHP treatment reduced the scratching times (two‐way ANOVAs for “time x genotype” or “time x treatment”; posthoc analyses for each time point using Holm‐Šidák adjustment; *P <* *0.05). B, Time courses (mean ± SD) of the scratching times after dermal injection of HCQ, which causes non‐histamine‐evoked itch via activation of Mas‐related G‐protein coupled receptors. Data are of n = 14 mice per genotype and n = 10 for DAHP vs vehicle. The scratching behaviour was reduced in LysM‐GCH1^−/−^ mice. It was increased in LysM‐GCH1‐HA mice, and was reduced after treatment with DAHP (two‐way ANOVAs for “time × genotype” or “time × treatment”; posthoc analyses for each time point using Holm‐Šidák adjustment; *P <* *0.05). C, Scatter plots showing the total scratching times of individual mice in the Cp48/80 and HCQ evoked itching models. The line is the mean and whiskers are the SD. The scratching behaviour differed significantly between groups (one‐way ANOVA separately for Cp48/80 ad HCQ, posthoc comparison of selected pairs, that is the respective control group, Šidák adjustment for multiple testing; *P < 0.05)

LysM‐GCH1^−/−^ mice showed reduced scratching behaviour as compared with the floxed controls in both itch models (Figure 4A, B time courses, Figure 4C total scratching time). Oppositely, scratching was increased in LysM‐GCH1‐HA mice, but only after HCQ stimulation (Figure 4A‐C). DAHP treatment, that is inhibition of GCH1, provided stronger suppression of the scratching responses in both itch models (Figure 4A‐C). DAHP does not affect motor functions or behavioural readouts of anxiety or depression at the doses used in the present study.^9^ The data suggested that BH4 not only controls mediator release by immunocytes, but is released itself and directly acts on pruriceptive neurons, in analogy to its functions in nociceptive neurons. Particularly, the enhancement of HCQ‐evoked pruritogenic effects in the LysM‐GCH1‐HA mice suggested that these mice have higher extracellular BH4, which acts as a co‐stimulant of HCQ. The latter is believed to act mainly through activation of sensory neurons.^20^, ^47^

### 3.4 Mast cell functions and BH4 stimulated calcium fluxes in sensory neurons

**Figure 5:**
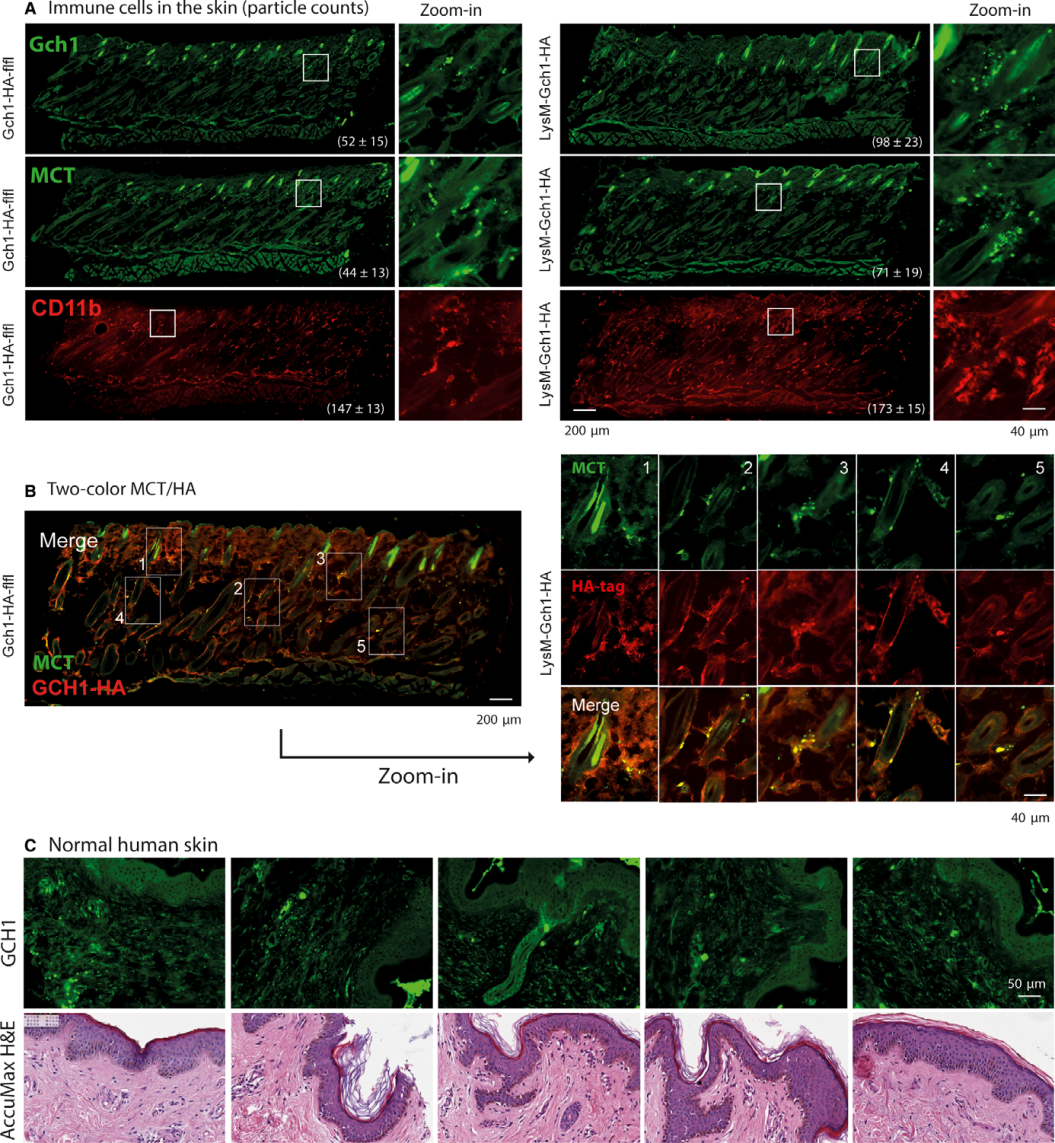
A, Immunofluorescent images showing GCH1‐positive myeloid cells (anti‐GCH1), mast cell tryptase (MCT) positive cells and CD11b positive myeloid cells in the skin of LysM‐GCH1‐HA and floxed control mice. The numbers show the counts of immunoreactive particles (mean ± SD) of n = 3 per group, counted using the Particle Counter implemented in ImageJ. The numbers of immunoreactive particles were significantly higher in the skin of LysM‐GCH1‐HA mice as compared with controls (two‐tailed unpaired *t* tests). Hair follicles appear to be positive for GCH1, which is in agreement with a previous study showing that hair follicles are a source of pterins.^64^ The white rectangles indicate the regions used for the zoom‐in images in the right columns. B, Immunofluorescent images showing two‐colour immunofluorescence analyses using anti‐MCT (green) and anti‐HA tag (red) in the skin of LysM‐GCH1‐HA mice. The right panel shows zoom‐in images of the rectangles from the overview in the left to reveal examples of double‐positive cells for GCH1‐HA (red) and MCT (green). C, Immunofluorescence analysis of GCH1 (green) in normal human skin using AccuMax tissue array. The bottom panel shows the H&E histology of the respective subjects of an adjacent section as provided by the manufacturer. Scale bars are indicated in the images

The results pointed to specific functions of BH4 in the context of itch rather than inflammation. To assess underlying peripheral mechanisms, we assessed mast cell degranulation and sensitivity of sensory neurons towards BH4. Immunofluorescence analyses revealed higher numbers of mast cells and macrophages in the skin in LysM‐GCH1‐HA mice (Figure 5A), suggesting that GCH1 overexpression per se resulted in a “pro‐itch” skin reaction (Figure 5A), providing a high source for high extracellular BH4. Mast cell tryptase (MCT), a marker for mast cells, was co‐expressed with GCH1‐HA (anti‐HA) in the skin of LysM‐GCH1‐HA mice (Figure 5B). In the normal human skin (Figure 5C), which we investigated to assess the translational aspect, GCH1 was expressed in endothelial cells and in immune cells with a morphology compatible with mast cells.

**Figure 6:**
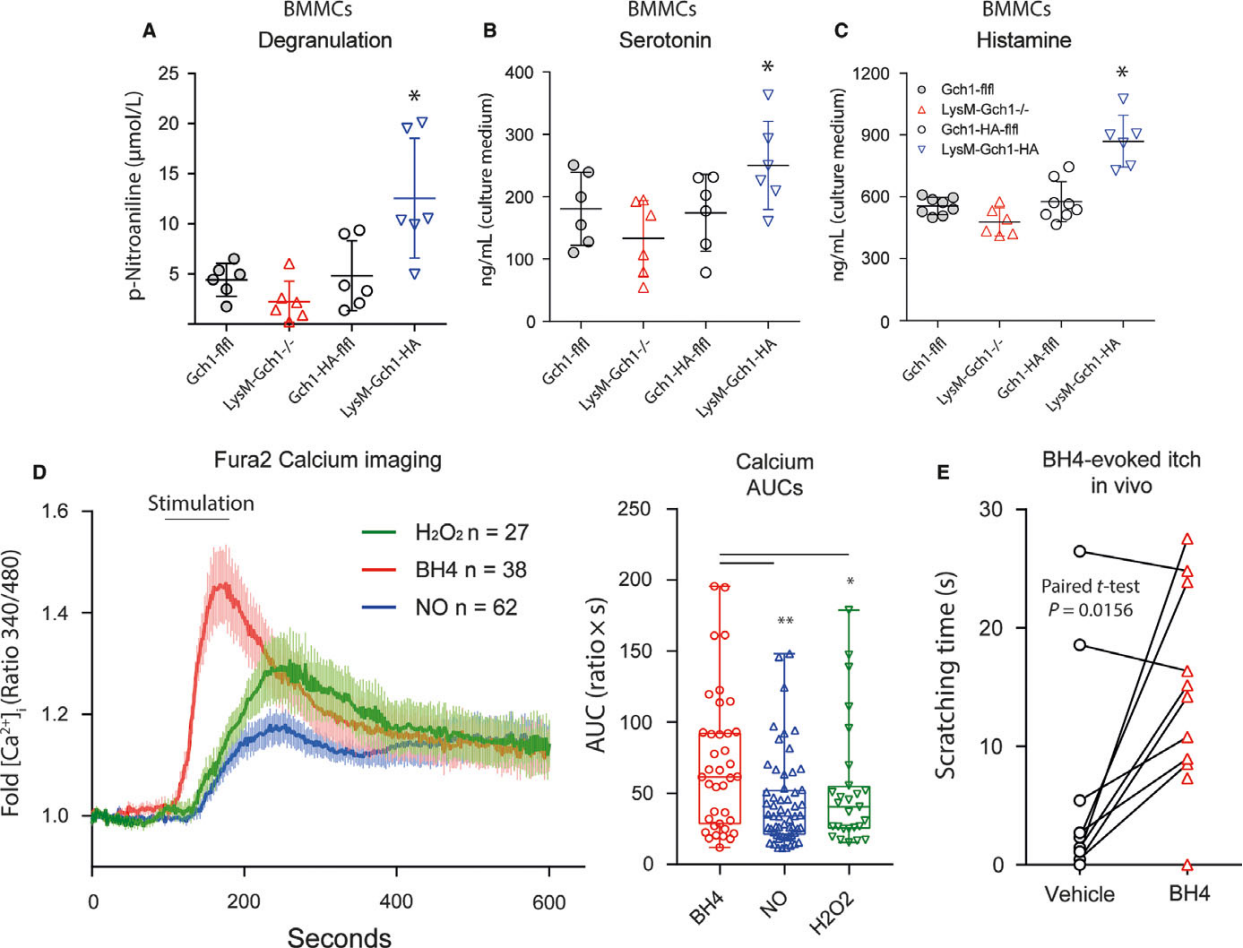
A‐C, Degranulation (A), serotonin (B) and histamine (C) release from bone marrow derived mast cells of LysM‐GCH1^−/−^ and GCH1‐flfl mice, and of LysM‐GCH1‐HA and GCH1‐HA‐flfl mice. The degranulation assay is based on analysis of a tryptase sensitive p‐nitroaniline substrate. Histamine and serotonin were analysed per ELISAs. Data are of six mice per group and duplicate analyses of mast cell cultures. Degranulation, histamine and serotonin release were increased in LysM‐GCH1‐HA mast cells (one‐way ANOVA, posthoc analysis of selected pairs, ie, the respective genotype control, Šidák adjustment for multiple testing; *P <* *0.05). D, Calcium imaging of primary sensory neurons of the dorsal root ganglia (DRGs) or adult naïve flfl mice upon stimulation with BH4 (100 μmol/L), the NO‐donor DEA‐NO (500 μmol/L) and (H_2_O_2_, 1 mmol/L). Intracellular [Ca^2+^]i was assessed fluorimetrically as absorbance ratio at 340 and 380 nm excitation (F340/380). Baseline ratios were recorded for 100 seconds before perfusion with the stimulants. The curves show the means ± SD of 27‐62 neurons (n number as indicated) and the right panel shows box plots of the area under the curve above baseline. The box represents the interquartile range, the line is the median and the whiskers show minimum to maximum. The scatters show results of individual neurons. The data are from neurons of four mice per stimulus. BH4 caused stronger and faster calcium fluxes than the other two (one‐way ANOVA, posthoc analysis with Šidák adjustment for multiple testing; *P <* *0.05, **P < 0.01). E, Scratching response upon intradermal injection of BH4 (100 μL, 100 μmol/L) or vehicle. Scratching behaviour was observed for 10 minutes after injection. Each mouse was subjected to both experiments, and mice were randomly allocated to first‐vehicle or first‐BH4 groups. The interval between experiments was 1 week. BH4 evoked significant scratching as revealed by paired, two‐sided *t* test, *P *=* *0.0156

Tetrahydrobiopterin availability in LysM‐GCH1^−/−^ mast cells was sufficient to maintain mast cell degranulation and serotonin and histamine release (Figure 6A‐C), because the respective enzymes use BH4 recycled from BH2 via QDPR. However, degranulation and release of the biogenic amines were significantly increased in LysM‐GCH1‐HA overexpressing mast cells (Figure 6A‐C). Hence, high levels of BH4 stimulated the release of mediators from mast cells, which is in line with previous studies showing that mast cell stimulation and degranulation involves phosphorylation, hence activation of GCH1.^17^

To assess if BH4, which is released from surrounding cells in the skin including immune cells and keratinocytes, directly activate sensory neurons including itch‐sensing neurons, we used Fura‐2 calcium imaging in primary DRG neurons (Figure 6D). Tetrahydrobiopterin caused robust calcium influxes and had stronger effects than the NO‐donor, DEA‐NO or H_2_O_2_, which agrees with our previous studies.^9^, ^11^ The concentrations of 100 μmol/L are in the range of biopterin plasma and tissue levels, suggesting that itch‐relevant concentrations occur in vivo. Indeed, intradermal injection of BH4 (100 μL, 100 μmol/L) evoked scratching responses in vivo in mice, which lasted for about 10 minutes (Figure 6E). The result is consistent with the hypothesis that BH4 directly activates sensory nerve terminals of itch‐sensing neurons in the skin and fortifies pro‐itch effects of pruritogens.

## 4 DISCUSSION

Inhibition of GCH1 with DAHP reliably reduces nociceptive hypersensitivity in rodent models of acute and chronic inflammatory and neuropathic pain,^9^, ^24^, ^35^ and genetic polymorphisms contribute to chronic pain in humans.^9^ Specific knockout of GCH1 in nociceptive sensory neurons partly replicates this phenotype,^25^ but overall effects are weaker, suggesting that DAHP additionally acts through non‐neuronal cells. The present study therefore assessed if and how BH4 from myeloid immune cells contributes to BH4‐dependent pain, inflammation and itch, either by changing enzyme functions and mediator release from these cells or by being released itself to act on neighbouring neurons.

Immune cells of myeloid origin contribute to the maintenance of nociceptive hypersensitivity and inflammation^48^, ^49^ and upregulate GCH1 on pro‐inflammatory stimulation.^33^, ^35^ Likewise, mast cells upregulate and phosphorylate GCH1 on sensitization with IgE,^13^, ^17^ but the functional in vivo implications of the latter have not been studied before. For inflammation, upregulation of GCH1 ensures that BH4‐dependent enzymes, particularly iNOS^50^ and AGMO,^37^ can function properly. While iNOS is a marker of pro‐inflammatory macrophages, AGMO upregulation is associated with polarization towards the M2‐like “inflammation‐resolving” phenotype.^37^ Hence, myeloid BH4 has dual functions in inflammation, and explains why neither knockout nor overexpression of GCH1 in lysozyme M positive immune cells affected inflammatory nociception in the present study.

**Figure 7:**
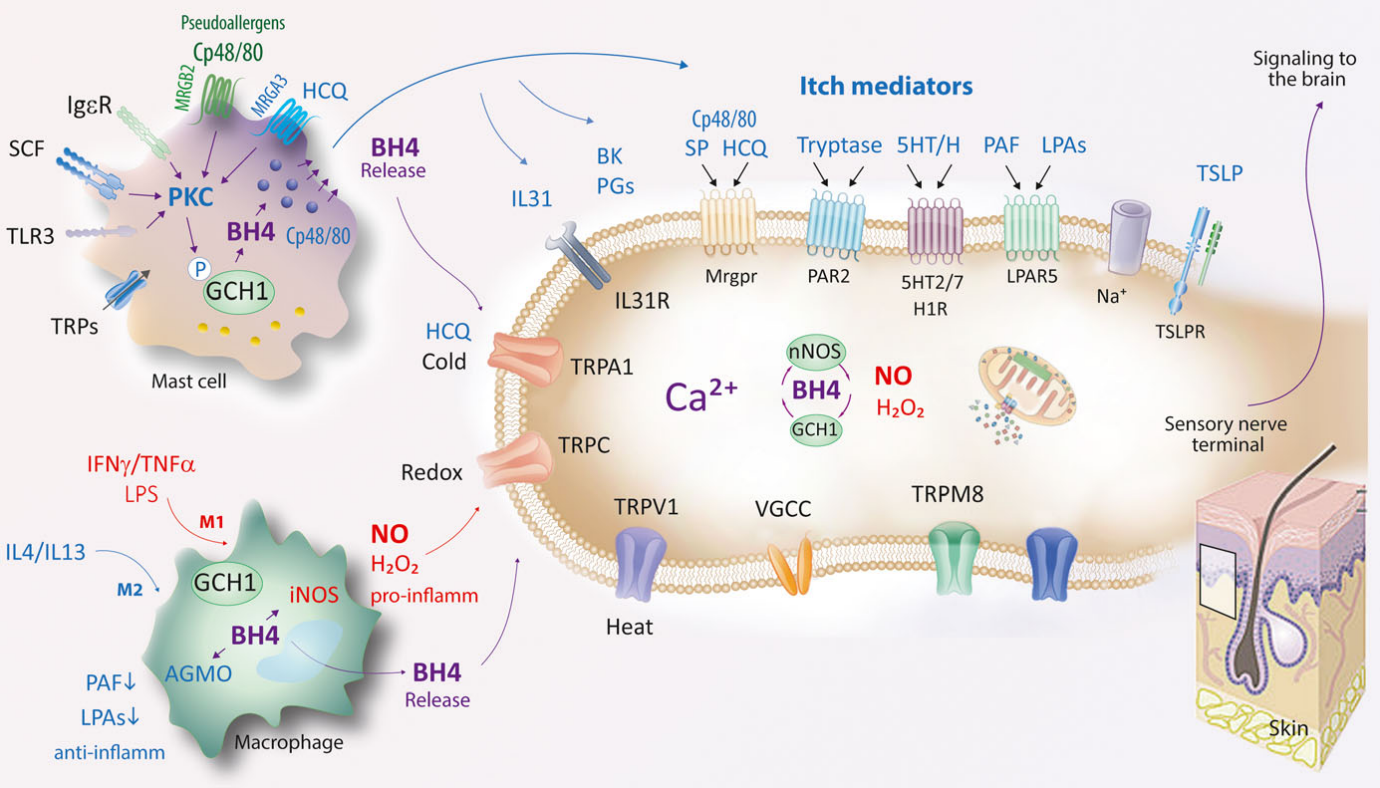
Molecular mechanisms of myeloid cell derived tetrahydrobiopterin (BH4) in inflammation and itch: signalling at the sensory nerve terminal. Mast cells, keratinocytes and lymphocytes release endogenous mediators upon specific stimulation, which activate itch‐responsive sensory neurons through specific receptors or ion channels including receptors for histamine, serotonin (5HT), substance P (SP), bradykinin (BK), platelet activating factor (PAF), lysophosphatidic acids (LPA), tryptase and other proteases, cytokines (eg, interleukin IL31, IL13, thymic stromal lymphopoietin TSLP) and arachidonic acid metabolites. Exogenous stimuli mimic the response. Compound 48/80 causes mast cell degranulation and activates a mast cell specific Mas‐related G‐protein coupled receptor, MrgprB2 (MRGB2), which is also activated by some drugs causing pseudoallergic reactions. Hydroxychloroquine (HCQ) activates Mas‐related G‐protein coupled receptors (Mrgpr's) of pruriceptive sensory neurons and of mast cells, the latter leading to mast cell degranulation. Mast cell stimuli for example agonists of Mrgpr's, Toll‐like receptors (TLR), transient receptor potential (TPR) channels and IgE converge on activation of protein kinase C (PKC), which then phosphorylates and activates GCH1 leading to de novo BH4 synthesis. BH4 in turn promotes mast cell degranulation and release of BH4 itself, the latter contributing to itch sensation via direct stimulation of calcium influx in sensory neurons. Hence, BH4 has dual pro‐itch effects (a) as a pro‐secretory signal within the releasing cell and (b) as a co‐itch mediator—ie likely as a pruritogen itself. BH4 also activates nociceptive neurons, that is it is not specific for pruriceptors. During inflammation, polarization of macrophages towards M1‐like pro‐inflammatory phenotypes are associated with upregulation of inducible nitric oxide synthase (iNOS), which requires BH4 to produce nitric oxide (NO). If the demand of BH4 is not (fully) met, iNOS produces H_2_O_2_ leading to oxidative stress, additional tissue damage and perpetuation of the inflammation. On the other hand, BH4 is required for metabolic functions of AGMO, whose upregulation is associated with M2‐like polarization, and leads to resolution of inflammation. Hence, BH4 within and from macrophages has opposing pro‐ and anti‐inflammatory effects, so that inflammatory pain phenotypes of myeloid GCH1 knockouts or overexpressors were indistinguishable from controls. AGMO, alkyglycerol monooxygenase; LPAR5, lysophosphatidic acid receptor 5; MRGPR, Mas‐related G‐protein coupled receptor; NGF, nerve growth factor; PLC, phospholipase C; TRL3, toll‐like receptor 3; TRPV, TRPC, TRPA, TRPM, transient receptor potential channels of V, C, A and M families; VGCC, voltage gated calcium channel

LysM‐Cre is a strong Cre‐driver, and the BMDM studies show that the recombination and functional changes of GCH1 were as expected. We infer that the mouse models were adequate. Hence, BH4‐dependent inflammatory nociception is not affected by myeloid BH4 but driven by the neuron's own GCH1/BH4 system, as revealed in our previous studies, in which GCH1 was knocked out specifically in sensory neurons.^25^ During inflammation, mice may either suffer or profit from increased GCH1‐mediated BH4 de novo production in myeloid immune cells. This duality is also found in cardiovascular disease models^51^, ^52^ and likely depends on alternative polarization towards pro‐ or anti‐inflammatory myeloid cells (Illustration Figure 7).

In the next set of experiments we focused on pruriception because of the prominent functions of GCH1 and BH4 in the skin.^2^, ^53^ Our results reveal that GCH1‐derived myeloid BH4 is a regulator of pruritogen‐evoked itching responses, raising the intriguing question of its specific functions in the context of allergy or skin diseases. GCH1 knockout reduced, and overexpression increased the scratching response in Cp 48/80‐evoked and hydroxychloroquine‐evoked pruritus, which are models for “histamine‐evoked” and “histamine‐independent” itch. HCQ effects are mediated through Mas‐related G‐protein coupled receptors (Mrgpr's)^47^ and TRP channels.^21^ Compound 48/80 acts via activation of mast cell specific, MrgprB2^40^ and by direct stimulation of mast cell degranulation^31^, ^40^and release of prurigenic mediators including proteases, histamine and serotonin (Figure 7). Tetrahydrobiopterin is coenzyme of tryptophan hydroxylase (TPH) and peripheral serotonin levels are sensitive to BH4 availability.^54^ TPH also uses BH4, which is recycled from BH2 via the QDPR salvage pathway. Therefore, baseline serotonin release from BMMCs was fairly maintained even in the absence of GCH1, and serotonin per se does not (fully) explain the attenuation of itching in LysM‐GCH1^−/−^ mice.

The GCH1 overexpressing mice however, presented with a strong enhancement of mediator release, which was accompanied by intensified scratching in the HCQ model, showing that particularly HCQ‐evoked itch was sensitive to high levels of surrounding BH4. We would have also expected an increase of Cp48/80 evoked scratching in the overexpressing mice, which might have failed because Cp48/80 per se causes maximum effects, which just remain at maximum. It is of note that the numbers of myeloid cells in the skin of LysM‐GCH1‐HA mice were increased, presumably providing an increased source of extracellular BH4, which was then immediately available to fortify HCQ's effects on sensory neurons, but may also promote pro‐inflammatory skin diseases. HCQ‐evoked itch is in part mediated through activation of Mrgpr's^47^ and TRPA1 channels of sensory neurons.^12^, ^55^ The latter are highly sensitive to oxidative modifications and therefore potential targets of BH4‐mediated strengthening of HCQ‐evoked pruriceptive effects. In addition, HCQ is known to cause mast cell degranulation of classical mediators and of phospholipid mediators.^45^, ^56^ Hence, in addition to GCH1 dependent degranulation of typical prurigenic mediators, release of BH4/NO per se appears to promote pruriceptor stimulation as a kind of co‐stimulant, which enhances effects of HCQ and other mediators on sensory nerve terminals.

The idea of a BH4 release is supported by previous studies showing concomitant upregulation of GCH1 and BH4‐dependent tryptophan hydroxylase in mast cells on stimulation with IgE.^17^ This was associated with the release of biogenic amines and NO,^38^, ^57^ which in turn is able to modify calcium entry by direct S‐nitrosylation of TRP channels.^58^ Our studies in BMDMs and BMMCs confirm that overexpression of GCH1 enhances BH4, NO and H_2_O_2_ release and mast cell degranulation, but the downstream path from BH4 to degranulation is still unknown. It has been shown that balances of NO/H_2_O_2_ impact on the release of biogenic amines from mast cells in itching models^59^ suggesting a redox dependent mechanism. It is noteworthy that protein kinase C dependent degranulation involves phosphorylation, hence activation of GCH1^17^ suggesting that BH4 is produced on PKC activation to replenish the mediator content of secretory vesicles.

Like in previous studies, we found that inhibition of GCH1 with DAHP had stronger effects than cell‐type specific GCH1 knockout. We infer that DAHP was not only acting through GCH1 inhibition in myeloid cells but also keratinocytes, which contribute to pruritogen release,^41^, ^60^, ^61^ and on itch‐sensing neurons or central neurons of the itch signalling pathways. In support of this idea, we observed that BH4 stimulation of primary sensory neurons evokes robust calcium currents, which agrees with previous experiments^9^, ^25^ and may involve redox modifications of itch‐ and nociception‐sensitive calcium channels. The expression pattern of specific calcium channels or receptors in BH4 responsive vs non‐responsive neurons may help to elucidate in the future for example by single cell RNA analyses, why pruriception was particularly sensitive to GCH1/BH4. Alternatively, reporter mouse lines like Runx1‐GFP^62^ or Ret‐GFP^63^ may help to identify BH4 susceptible sensory neuron subtypes.

In summary, we show that GCH1‐mediated BH4 synthesis in myeloid cells promotes itch in histamine‐dependent and independent models involving regulations of the release of biogenic amines, BH4, NO and H_2_O_2_ and subsequent effects on neurons. Indeed, intradermal BH4 provoked scratching behaviour in mice suggesting that BH4 contributes to itch sensation.

## ACKNOWLEDGEMENTS

We thank Sandra Labocha and Yannick Schreiber for biopterin analyses and Michael Costigan for providing the GCH1‐HA‐floxed mice.

## CONFLICT OF INTEREST

The authors declare no conflict of interest. The funding sponsors had no role in the design of the study; in the collection, analyses or interpretation of data; in the writing of the manuscript, and in the decision to publish the results.

## AUTHOR CONTRIBUTION

KZ and CF performed experiments and analysed data; AH maintained mouse colonies and provided experimental advice; KC generated GCH1‐floxed mice; KW provided AGMO‐specific tools and gave experimental advice; GG organized the analytical laboratory; IT conceived and organized the study, performed live imaging experiments, analysed data, generated the figures and wrote the manuscript. All authors contributed to drafting or editing of the manuscript.
