# Supplementary material for: Mast cell tetrahydrobiopterin contributes to itch in mice
Source: J Cell Mol Med. 2018 Nov 18;23(2):985–1000. doi: 10.1111/jcmm.13999 (PMC6349351; doi:10.1111/jcmm.13999)
Supplement: Supplementary file 1 [file JCMM-23-985-s001.pdf]

Supplementary information

Suppl. Table 1: Antibodies for Flow Cytometry (FACS), immunohistochemistry and western blot

Antibody	Host	Species reactivity	Vendor	Product#	Dilution	Method
β-actin	rabbit	mouse	Sigma	A2066	1:1,000	WB
β-actin	mouse	mouse	Sigma	A5441	1:1,000	WB
CD117-PE	rat	mouse	Miltenyi Biotec	130-102-795	1:50 (IHC)	IHC, FACS
CD11b-FITC	rat	mouse	Serotec	MCA711	1:250	IHC
DAPI	-	-	AppliChem	A1001	1:1,000	IHC
FcεRIα-FITC	hamster	mouse	Biolegend	134305	1:50 (IHC)	IHC, FACS
GCH1	mouse	human	Abnova	H00002643-M01	1:200	IHC
GCH1	rat	human, mouse	Sigma	SAB4200046	1:500	WB
HA-Tag	mouse	mouse	Cell Signaling	2367	1:500 (IHC)<br>1:1,000 (WB)	IHC, WB
Mast cell tryptase (MCT)	rabbit	human	Abcam	ab151757	1:300	IHC
AGMO	rabbit	human/mouse	Proteintech	21355-1-AP	1:200	IHC

Supplementary Figure 1

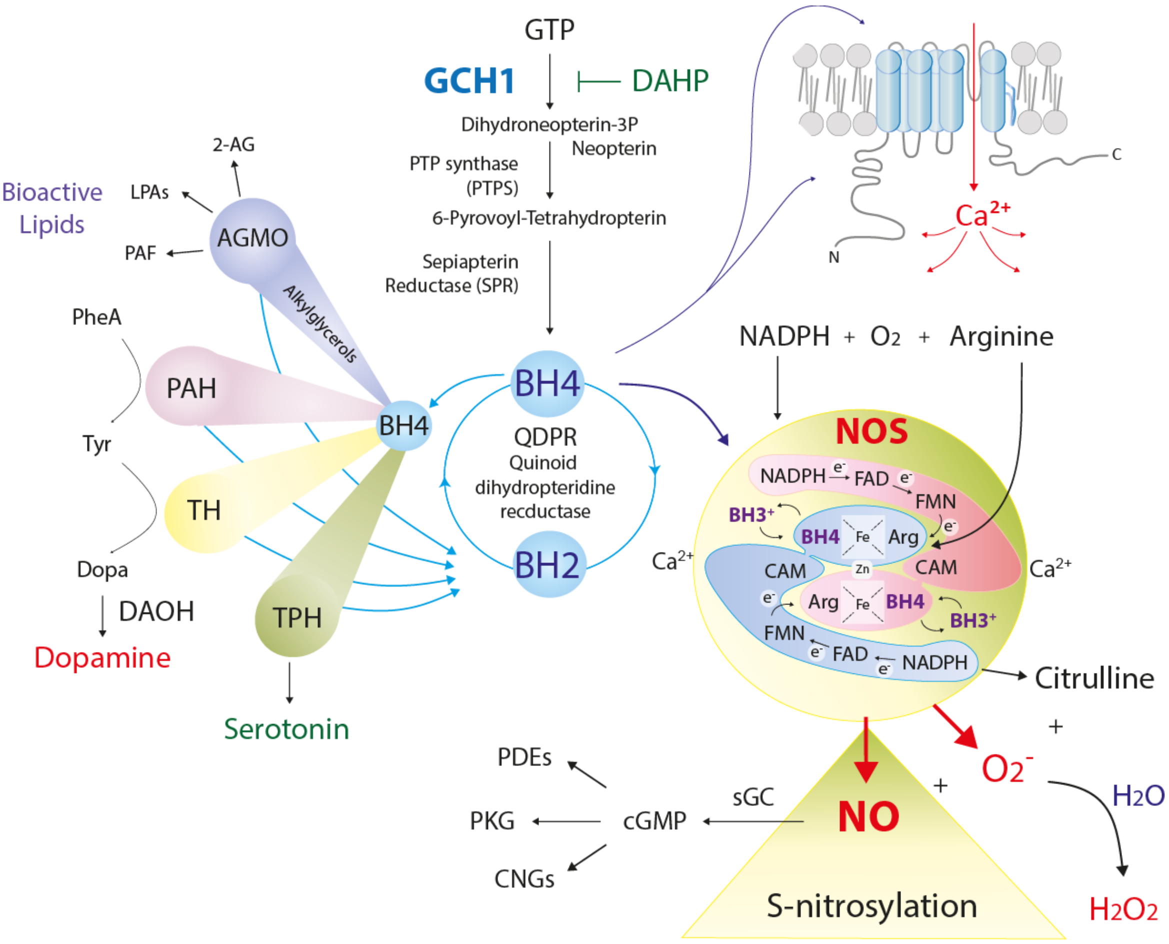

Production of and signaling of tetrahydrobiopterin and nitric oxide

Tetrahydrobiopterin (BH4) is produced from guanosine triphosphate (GTP) in a 3-step enzymatic cascade starting with the rate limiting GTP cyclohydrolase (GCH1), which converts GTP to dihydroneopterin-3-phosphate and neopterin, the latter a bypass product. In the final step, sepiapterin reductase (SPR) produces BH4, which is essential coenzyme for hydroxylases involved in biogenic amine synthesis, alkylglycerol monooxygenase (AGMO) and nitric oxide synthases (NOS). During the catalytic process of hydroxylases and AGMO BH4 is oxidized to BH2, which can be recycled with help of quinoid dihydropteridine reductase (QDPR). The hydroxylases, which dependent on BH4, are tyrosine hydroxylase (TH) for production of dopamine via the downstream enzyme, dopamine beta-hydroxylase (DBH), tryptophan hydroxylase (PAH) for production of serotonin and phenylalanine hydroxylase (PHA) for metabolism of phenylalanine. NOS enzymes directly regenerate BH4 within the catalytic site without need for further enzymes. BH4 or bioptern are released and can act on neighboring cells, and although BH4 is polar, it can be taken up, presumably via a pterin transporter. BH4 stimulates calcium currents in neurons likely via direct activation of calcium channels including TRP channels and voltage gated calcium channels. BH4 also elicits calcium dependent signals in immune cells.

There are three NOS isoenzymes with distinct cellular localization and regulation: neuronal NOS (NOS1), endothelial NOS3 and inducible NOS2, the latter is upregulated on inflammatory stimulation, but is also constitutively expressed in some tissues. The structure, composition and catalytic activities are similar. NOSs form homodimers, each monomer consisting in reduction and oxidation domains,

which are linked by a calmodulin binding domain that confers calcium sensitivity and facilitates the flux of electrons. The oxidation domain has a central heme group and binding sites for the substrate L-arginine and the coenzyme BH4. NOSs oxidize the substrate L-arginine in the presence of oxygen (O2) and NADHP to produce citrulline, nitric oxide and superoxide (O2-). This reaction essentially requires BH4, which undergoes an oxidation-reduction cycle to reconstitute heme iron (Fe^{2+}), which is oxidized during the process (Fe^{3+}). In case of a relative deficiency of BH4 the redox cycles get uncoupled resulting in high production of superoxide rather than NO. Superoxide reacts with H_2O to form hydrogen peroxide (H_2O_2), which has signaling functions but may also produce oxidative stress. NO activates soluble guanylylcyclase (sGC), leading to the production of cyclic GMP and activation of protein kinase G (PKG). NO causes direct reversible S-nitrosylation of a number of proteins including glutamate receptors, calcium channels, redoxins, caspases, beta arrestins, calmodulin, transport proteins and chaperones. These SNO modification cause subtle adjustments of the proteins' functions, interactions, traffic or metabolism.

Suppl. Figure 2a and b: Full Western Blots

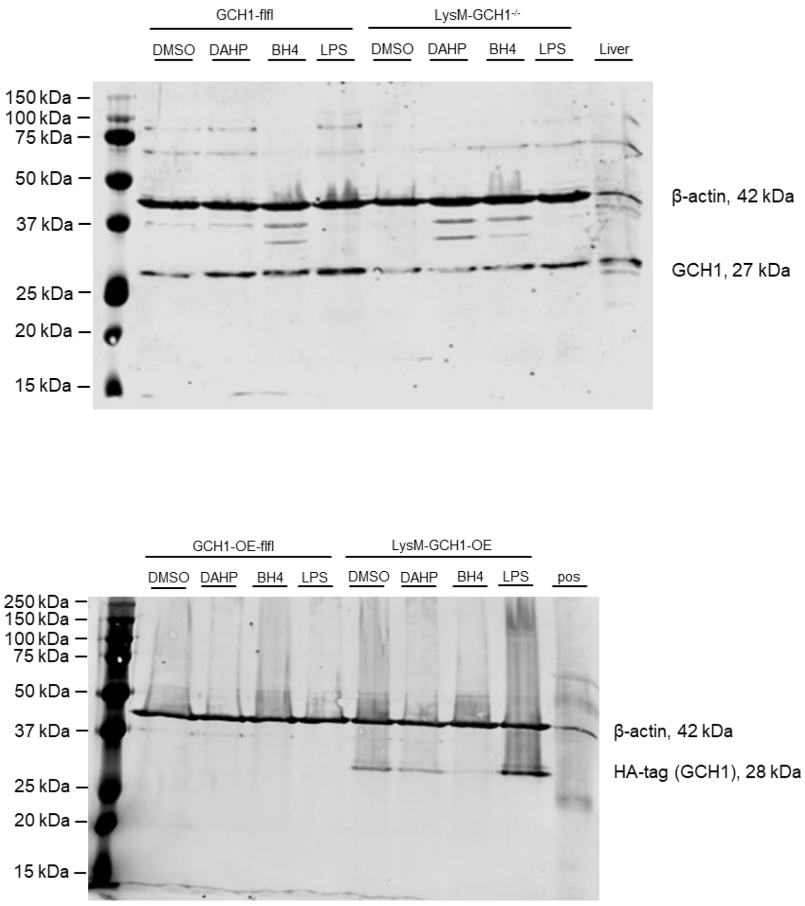

Suppl. Fig. 2a: Full, uncut Western Blots of Figure 1C of GCH1 protein expression in BMDMs of LysM-Gch1$^{-/-}$ (knockout upper) and LysM-Gch1-HA mice (overexpression with HA-tag; bottom) and the respective floxed control mice as shown in Figure 1C. Liver was used as control tissue (last lane). The LysM-Gch1-HA blot was developed with anti-HA tag antibody.

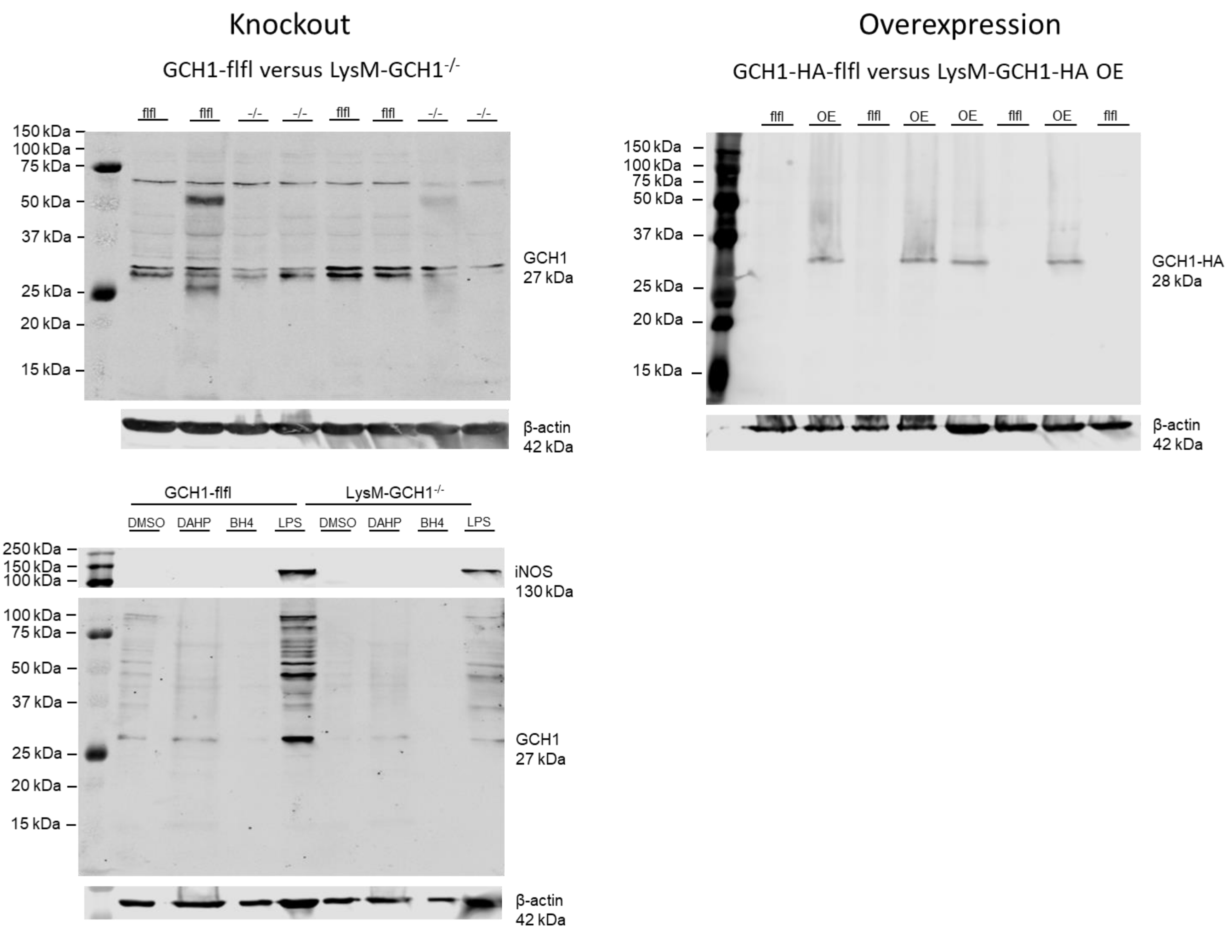

Suppl. Figure 2b: Further Western Blots showing the LysM-Cre mediated deletion or overexpression of GCH1, not included in the main body of the manuscript
